# Stroke etiologies in patients with COVID-19: the SVIN COVID-19 multinational registry

**DOI:** 10.1186/s12883-021-02075-1

**Published:** 2021-01-30

**Authors:** María E. Ramos-Araque, James E. Siegler, Marc Ribo, Manuel Requena, Cristina López, Mercedes de Lera, Juan F. Arenillas, Isabel Hernández Pérez, Beatriz Gómez-Vicente, Blanca Talavera, Pere Cardona Portela, Ana Nuñez Guillen, Xabier Urra, Laura Llull, Arturo Renú, Thanh N. Nguyen, Dinesh Jillella, Fadi Nahab, Raul Nogueira, Diogo Haussen, Ryna Then, Jesse M. Thon, Luis Rodríguez Esparragoza, Maria Hernández-Pérez, Alejandro Bustamante, Ossama Yassin Mansour, Mohammed Megahed, Tamer Hassan, David S. Liebeskind, Ameer Hassan, Saif Bushnaq, Mohamed Osman, Alejandro Rodriguez Vazquez, SVIN Multinational Registry and Task Force, Patricia Feineigle, Mohamad Abdalkader, Sergio Amaro, Hugo Aparicio, Ivo Bach, Jordi Blasco, Ángel Chamorro, Judith Clark, Alexandra Czap, Natalia Perez de la Ossa, Shashvat Desai, Laura Dorado, Denise Evans, Mudassir Farooqui, Meritxell Gomis, Mark Heslin, Chris Higham, Ashutosh P. Jadhav, Tudor G. Jovin, Artem Kaliaev, Priyank Khandelwal, Rakeshh Khatri, Amy Krueger, Carlos Laredo, Italo Linfante, Antonio López, Racheal McCoy, Mònica Millàn, Mahmoud H. Mohammaden, Leigh Moore, Isaac Nuño Ruiz, Víctor Obach, Darko Quispe Orozco, Santiago Ortega-Gutierrez, Pratit Patel, Mary S. Patterson, Gonzalo Valle Peñacoba, Leonardo Pisani, Laurie Preston, Razvan Alexandru Radu, Vivek Rai, Anna Ramos-Pachón, Ankit Rana, Srikant Rangaraju, Jose Rafael Romero, Salvatore Rudilosso, Emma Sanborn, Sunil Sheth, Julie G. Shulman, Amit Singla, Ainsley Smith, Amy Starosciak, Lauren Thau, Ephrem Teklemariam, Elena Oana Terecoasa, Cristina Tiu, Vlad Eugen Tiu, Israr Ul Haq, Martha Vargas, Víctor Vera, Osama Zaidat, Cynthia Zevallos, Alicia M. Zha

**Affiliations:** 1 Institute of Biomedical Research of Salamanca, Department of Neurology, Hospital Universitario de Salamanca, Salamanca, Spain; 2 Department of Neurology, Hospital Clínico Universitario de Valladolid, Valladolid, Spain; 3 Cooper Neurologic Institute, Cooper University Hospital, Camden, NJ USA; 4 Stroke Unit, Department of Neurology, Vall d’Hebron Research Institute, Barcelona, Spain; 5 Departament of Medicina, Universitat Autónoma de Barcelona, Barcelona, Spain; 6 Department of Neurology, Hospital Universitari, Bellvitge, Barcelona, Spain; 7 Department of Neurology, Hospital Clínic, Barcelona, Spain; 8 Department of Neurology, Department of Radiology, Department of Neurosurgery, Boston Medical Center, Boston University School of Medicine, Boston, MA USA; 9 Department of Neurology, Emory University School of Medicine, Atlanta, USA; 10 Department of Neurology, Grady Memorial Hospital, Atlanta, GA USA; 11 Stroke Unit, Neuroscience Department, Hospital Universitari Germans Trias i Pujol, Badalona, Barcelona, Spain; 12 Neurology Department, Stroke and neurointervention unit, Alexandria University, Alexandria, Egypt; 13 Critical care Department, Alexandria University, Alexandria, Egypt; 14 Neurosurgery Department, Stroke and Neurointervention unit, Alexandria University, Alexandria, Egypt; 15 Department of Neurology, Ronald Reagan UCLA Medical Center, Los Angeles, USA; 16 Department of Clinical Neuroscience Research, Valley Baptist Medical Center, Harlingen, TX USA; 17 Department of Neurology, University of Texas Rio Grande Valley, Harlingen, TX USA; 18 Neuroscience Institute, Bon Secours Mercy Health St. Vincent Hospital, Toledo, OH USA

**Keywords:** Stroke, COVID-19, Coronavirus, Cryptogenic, Mortality

## Abstract

**Background and purpose:**

Coronavirus disease 2019 (COVID-19) is associated with a small but clinically significant risk of stroke, the cause of which is frequently cryptogenic. In a large multinational cohort of consecutive COVID-19 patients with stroke, we evaluated clinical predictors of cryptogenic stroke, short-term functional outcomes and in-hospital mortality among patients according to stroke etiology.

**Methods:**

We explored clinical characteristics and short-term outcomes of consecutively evaluated patients 18 years of age or older with acute ischemic stroke (AIS) and laboratory-confirmed COVID-19 from 31 hospitals in 4 countries (3/1/20–6/16/20).

**Results:**

Of the 14.483 laboratory-confirmed patients with COVID-19, 156 (1.1%) were diagnosed with AIS. Sixty-one (39.4%) were female, 84 (67.2%) white, and 88 (61.5%) were between 60 and 79 years of age. The most frequently reported etiology of AIS was cryptogenic (55/129, 42.6%), which was associated with significantly higher white blood cell count, c-reactive protein, and D-dimer levels than non-cryptogenic AIS patients (p</=0.05 for all comparisons). In a multivariable backward stepwise regression model estimating the odds of in-hospital mortality, cryptogenic stroke mechanism was associated with a fivefold greater odds in-hospital mortality than strokes due to any other mechanism (adjusted OR 5.16, 95%CI 1.41–18.87, *p* = 0.01). In that model, older age (aOR 2.05 per decade, 95%CI 1.35–3.11, *p* < 0.01) and higher baseline NIHSS (aOR 1.12, 95%CI 1.02–1.21, *p* = 0.01) were also independently predictive of mortality.

**Conclusions:**

Our findings suggest that cryptogenic stroke among COVID-19 patients carries a significant risk of early mortality.

**Supplementary Information:**

The online version contains supplementary material available at 10.1186/s12883-021-02075-1.

## Introduction

Since December 2019, coronavirus disease 2019 (COVID-19), caused by severe acute respiratory syndrome coronavirus 2 (SARS-CoV-2), has become a pandemic infecting more than 81 million people worldwide and causing more than 1.700.000 deaths [1]. Although respiratory symptoms are most commonly reported, neurological symptoms are increasingly recognized, and range from 36 to 56% according to large series of hospitalized patients [2–4], with a small but clinically significant risk of acute ischemic stroke (AIS) [5–7].

Potential mechanisms of SARS-CoV-2-associated stroke have been described and include a prothrombotic state [3, 8, 9], hyperinflammatory response, cardiomyopathy and endothelial injury from direct viral invasion [10–12]. Among hospitalized patients, COVID-19 has been recognized as an independent predictor of AIS and is associated with poor outcomes with considerable early mortality in small observational cohorts [13, 14]. The aim of this study was to characterize clinical, radiographic, and laboratory predictors of cryptogenic stroke using a multinational registry, and to evaluate short-term outcomes of patients with SARS-CoV-2 associated stroke according to traditional stroke mechanisms.

## Methods

### Study design and participants

Between 3/1/2020 and 6/16/2020, we pooled consecutive patients >/= 18 years of age hospitalized or evaluated in the emergency department (ED) with AIS and confirmed diagnosis of COVID-19 by oropharyngeal polymerase chain reaction (PCR) or IgG and/or IgM antibody in sera, using local commercial assays, in 31 hospitals across 4 countries. A more detailed summary of the Methods has been previously described [7]. Informed consent was waived by the local institutional review board at each participating center. No sample size calculations were made for this study as analyses were exploratory and hypothesis-generating. The results of this investigation are reported in accordance with the STrengthening the Reporting of OBservational studies in Epidemiology (STROBE) statement.

### Data collection

Demographic, clinical, and laboratory findings (including method of COVID-19 diagnosis), as well as stroke treatment (including intravenous thrombolysis, endovascular thrombectomy, and antithrombotic therapy), and short-term functional outcomes at time of discharge were collected. Stroke etiology was determined by local site investigators (vascular neurologists) using a modified Trial of ORG 10172 in Acute Stroke Treatment (TOAST) classification [15]. Cryptogenic stroke was defined as stroke of undetermined etiology after exclusion of cardiac sources of embolism, large artery atherosclerotic stenosis, and small vessel disease. Patients with incomplete workup were also considered to have cryptogenic infarcts. Pre-morbid and discharge functional status were determined using the modified Rankin Scale [16, 17]. Neuroimaging was performed at the discretion of the treating physician and radiographic findings (e.g., location of infarction, presence of an intracranial occlusion) were reported by local site investigators. Each center reported their in-hospital mortality rate among all COVID-19 patients throughout the study period. Data were recorded on a HIPAA-compliant, online registry, as previously described [18]. Data elements with < 50% completion rate (e.g., erythrocyte sedimentation rate) were not reported in order to limit selection bias.

### Outcome measures

The primary study outcome was the diagnosis of a cryptogenic (undetermined) mechanism of stroke. Secondary outcomes included discharge disability (according to mRS), discharge disposition, and in-hospital mortality among patients with COVID-19 when stratified by cause of acute ischemic stroke. To maximize specificity of findings, patients with more than one possible stroke mechanism (*n* = 9) were excluded from comparative analyses involving stroke mechanisms.

### Statistical analysis

Normality of continuous data was assessed histographically and confirmed using the Shapiro-Wilk test. Non-normally distributed continuous variables are summarized as medians with interquartile range. Categorical variables are presented as frequencies (%). Descriptive statistics were used to compare groups, with the χ^2^ test for discrete variables (or Fisher’s exact test when cell counts were 5 or less), and the Wilcoxon Rank-sum test or Kruskal-Wallis equality of populations rank test for non-normally distributed continuous variables, as appropriate. Bivariate analyses were performed to associate baseline variables with the occurrence of endpoints. Backward stepwise logistic regression was performed to estimate the association between clinical, laboratory, and radiographic findings with the primary and secondary outcomes. Estimates of association are presented as odds ratios (OR) and respective 95% confidence intervals (CI), with adjustment for all variables significant to *p* < 0.2 on the respective bivariate analysis. Variables were eliminated from each adjusted model if they were no longer significant to *p* < 0.2. All adjusted regression models were clustered by site. Using the remaining variables from the final backward stepwise regression model for mortality, we created a simple index assigning 1-point to each variable (age, male sex, history of diabetes, NIHSS, and cryptogenic stroke; Supplementary Table 1). In order to reduce overfitting, NIHSS was stratified such that one point was assigned for values ≥10 and age stratified such that one point was assigned if the patient were ≥ 60 years old based on visual association of these thresholds with respect to associated risk of mortality (Supplementary Figs. 1 and 2). The mortality rate of the cohort was assessed for each point value. A receiver operating characteristic curve was generated to test the association between the mortality score and in-hospital mortality. Model performance was assessed using the c-statistic with 95% CI. The statistical significance level of all tests was defined as a *p*-value ≤0.05. As this was an exploratory study, no adjustments were made for multiple comparisons. *P*-values are provided for conventional purposes only and should be interpreted with caution. All tests were performed at the two-sided level using STATA (College Station, TX) version 15.0. Missing data were not imputed. Fully de-identified data can be made available upon reasonable request of the corresponding author.

## Results

### Clinical features

Of the 14,483 laboratory-confirmed SARS-CoV-2 patients, 156 (1.1%) were diagnosed with a clinical and/or radiographic acute ischemic stroke. Antecedent systemic symptoms were reported with variable frequency but included fever in 69/134 patients (51%), 77/133 (58%) with cough, 60/128 (47%) dyspnea, 21/112 (19%) chest pain, 18/107 (17%) myalgias, 12/110 (11%) headache, and < 10% with symptoms of congestion, dizziness, odynophagia, hyposmia, or hypogeusia. Among stroke patients, 61/155 (39.4%) were female, 84/125 (67.2%) White, and more than half of patients (*n* = 88/143, 61.5%) were between 60 and 79 years of age. Seventeen of 145 patients (11.7%) received intravenous thrombolysis, and 14/156 (9.0%) were reported to experience any intracerebral hemorrhage—1 patient experienced a NIHSS worsening of 4 points attributed to a parenchymal hematoma, however this patient did not receive intravenous thrombolysis or thrombectomy.

The etiology of stroke was reported in 129 of 156 stroke patients (82.7%), with cryptogenic being commonly reported (*n* = 55, 42.6%). Compared to patients with any other defined stroke mechanism (or multiple possible mechanisms), patients with cryptogenic stroke were more frequently Hispanic (51.0% vs. 33.3%, *p* = 0.05), had less atrial fibrillation (*p* < 0.01) and congestive heart failure (*p* = 0.05; Table 1).

**Table 1:** Demographic data

	All patients acute ischemic stroke^a^ (*n* = 156)	Cryptogenic stroke (*n* = 55)	Cardioembolic stroke (*n* = 35)	Large vessel stroke (*n* = 15)	Small vessel stroke (*n* = 4)	Other (*n* = 11)	*p*-value^b^	*p*-value^c^
Age, no. (%)							0.10	0.97
< 30	0 (0)	0 (0)	0 (0)	0 (0)	0 (0)	0 (0)		
30–39	3 (2.5)	1 (1.8)	2 (5.7)	0 (0)	0 (0)	2 (18.2		
40–49	12 (10.0)	6 (10.9)	2 (5.7)	0 (0)	0 (0)	4 (36.4)		
50–59	15 (12.5)	6 (10.9)	5 (14.3)	2 (13.3)	0 (0)	2 (18.2)		
60–69	36 (30.0)	19 (34.6)	8 (22.9)	6 (40.0)	1 (25.0)	2 (18.2)		
70–79	37 (30.8)	16 (29.1)	13 (37.1)	5 (33.3)	2 (50.0)	1 (9.1)		
80–89	12 (10.0)	5 (9.1)	4 (11.4)	2 (13.3)	1 (25.0)	0 (0)		
> 89	5 (4.2)	2 (3.6)	3 (8.6)	0 (0)	0 (0)	0 (0)		
Sex, no. female (%)	61/155 (39.4)	19/55 (34.6)	15/35 (42.9)	5/14 (35.7)	0/4 (0)	8/11 (72.7)	0.08	0.45
Race, no. (%)							0.52	> 0.9
White	84/125 (67.2)	31/45 (68.9)	21/28 (75.0)	9/12 (75.0)	2/3 (66.7)	5/10 (50.0)		
Black	35/125 (28.0)	12/45 (26.7)	7/28 (25.0)	2/12 (16.7)	1/3 (33.3)	4/10 (40.0)		
Asian	2/125 (1.6)	1/45 (2.2)	0/28 (0)	0/12 (0)	0/3 (0)	0/10 (0)		
More than one race	2/125 (1.6)	1/45 (2.2)	0/28 (0)	1/12 (8.3)	0/3 (0)	0/10 (0)		
Other	2/125 (1.6)	0/45 (0)	0/28 (0)	0/12 (0)	0/3 (0)	1/10 (0)		
Hispanic ethnicity, no. (%)	58/140 (41.4)	25/49 (51.0)	10/33 (30.3)	4/12 (33.3)	4/4 (100)	10/11 (90.9)	0.02	0.05
Diagnosis of COVID-19 ^d^, no. (%)							0.11	0.83
Nasopharyngeal PCR	153 (98.1)	55/55 (100)	35/35 (100)	15/15 (100)	4/4 (100)	10/11 (90.9)		
Serum IgM and/or IgG	6 (3.8)	2/55 (3.6)	3/35 (8.6)	0/15 (0)	1/4 (25.0)	1/11 (9.1)		
Known COVID-19 exposure, no. (%)	19 (12.2)	8/55 (14.6)	5/35 (14.3)	1/15 (6.7)	0/4 (0)	2/11 (18.2)	0.82	0.69
Medical history, no. (%)								
Hypertension	111/154 (72.1)	38/53 (71.7)	26/35 (74.3)	12/15 (80.0)	4/4 (100)	4/11 (36.4)	0.10	0.87
Diabetes mellitus	65/152 (42.8)	22/51 (43.1)	13/35 (37.1)	5/15 (33.3)	2/4 (50.0)	7/11 (63.6)	0.56	0.87
Dyslipidemia	58/142 (40.9)	18/53 (34.0)	17/35 (48.9)	7/15 (46.7)	3/4 (75.0)	0/11 (0)	0.01	0.33
Atrial fibrillation	22/148 (14.9)	0/52 (0)	16/35 (45.7)	0/15 (0)	0/4 (0)	0/11 (0)	< 0.01	< 0.01
Congestive heart failure	27/154 (17.5)	5/53 (9.4)	9/35 (25.7)	4/15 (26.7)	0/4 (0)	1/11 (9.1)	0.17	0.05
Active tobacco use	15/145 (10.3)	4/52 (7.7)	5/33 (15.2)	0/15 (0)	1/4 (25.0)	0/11 (0)	0.21	0.68
Prior stroke	15/140 (10.7)	6/42 (12.5)	2/33 (6.1)	0/13 (0)	0/4 (0)	0/11 (0)	0.59	0.33
Chronic renal insufficiency (stage III/IV or dialysis-dependent)	16/144 (11.1)	3/47 (6.4)	1/33 (3.0)	2/13 (15.4)	0/4 (0)	3/11 (27.3)	0.11	0.49
Chronic obstructive pulmonary disease and/or asthma	11/135 (8.2)	2/48 (4.2)	3/33 (9.1)	0/13 (0)	1/4 (25.0)	0/11 (0)	0.28	0.24
Cancer	8/134 (6.0)	4/47 (8.5)	1/33 (2.9)	0/13 (0)	1/4 (25.0)	0/11 (0)	0.29	0.35
Premorbid mRS, median (IQR)	0 (0–1) (*n* = 124)	0 (0–0) (*n* = 49)	0 (0–1) (*n* = 33)	0 (0–0) (*n* = 13)	0 (0–1) (*n* = 4)	0 (0–0) (*n* = 10)	0.54	> 0.9

*Of the 11 patients with ‘Other’ etiologies, 2 had hypercoagulable states due to malignancy, 1 with hypercoagulability on laboratory testing unrelated to malignancy, 2 with arterial dissection, 1 with vasculitis due to mucormycosis, 3 with radiographic findings of posterior reversible encephalopathy with infarction, and 2 with etiologies not otherwise specified*

^a^ Acute ischemic stroke includes suspected or radiographically-confirmed cases, and in patients with multiple possible stroke mechanisms (*n* = 9). Patients with multiple stroke mechanisms were not included in the subsequent columns in order to minimize heterogeneity

^b^ *P*-values indicate categorical comparisons between all stroke subtypes

^c^ *P*-values indicate comparisons between cryptogenic and non-cryptogenic stroke subtypes (including strokes due to multiple possible etiologies)

^d^ 3 patients with an acute ischemic stroke were diagnosed with COVID-19 using both serum antibodies and nasopharyngeal PCR. COVID-19 denotes coronavirus disease 2019, PCR polymerase chain reaction

There was no difference with respect to age, sex, race, or history of pre-morbid disability among patients with cryptogenic versus known stroke mechanisms.

In a separate analysis of 11 sites included in this study which submitted data of ischemic stroke patients irrespective of SARS- CoV-2 infection during a similar period the year prior (3/1/19–05/31/19), the prevalence of cryptogenic (unspecified or undetermined) stroke was 19.7% (260/1319 patients) [19]. Using a two-sample test of proportions, the rate of cryptogenic stroke was significantly higher in this population of COVID-19-associated stroke (42.6% vs. 19.6%, *p* < 0.001). Furthermore, the reported in-hospital mortality rate among these patients was also significantly greater than in this historic control group (38.1% vs. 7.8%, *p* < 0.001).

On admission, neurological deficits according to baseline NIHSS were generally moderate-to-severe among all patients, with more severe deficits noted in patients with strokes due to a cryptogenic mechanism, cardioembolism, or other identifiable mechanism, compared to patients with large or small vessel disease (*p* = 0.02; Table 2).

**Table 2:** Clinical, laboratory, and radiographic measures and management

	All patients acute ischemic stroke^a^ (*n* = 156)	Cryptogenic stroke (*n* = 55)	Cardioembolic stroke (*n* = 35)	Large vessel stroke (*n* = 15)	Small vessel stroke (*n* = 4)	Other (*n* = 11)	*p*-value^b^	*p*-value^c^
**Clinical and radiographic findings**								
Baseline NIHSS median, (IQR)	13 (5–21) (*n* = 130)	12 (6–23) (*n* = 48)	14 (6–21) (*n* = 32)	8 (4–11) (*n* = 14)	2 (1–2) (*n* = 4)	21 (9–22) (*n* = 10)	0.02	0.53
Head CT performed, no. (%)	135/151 (89.4)	48/55 (87.3)	34/35 (97.1)	14/15 (93.3)	4/4 (100)	10/11 (90.9)	0.56	0.25
First CT indicating acute stroke, no. (%)	76/132 (57.6)	33/48 (68.8)	21/34 (61.8)	10/14 (71.4)	0/4 (0)	4/10 (40.0)	0.04	0.10
CT angiogram performed, no. (%)	99/151 (65.6)	36/55 (65.5)	23/35 (65.7)	14/15 (93.3)	4/4 (100)	10/11 (90.9)	0.07	0.36
Intracranial occlusion, no. (%)	53/107 (49.5)	17/37 (46.0)	17/24 (70.8)	6/14 (42.9)	0/4 (0)	3/10 (30.0)	0.04	0.70
MRI brain performed, no. (%)	55/151 (36.4)	21/55 (38.2)	11/35 (31.4)	5/15 (33.3)	1/4 (25.0)	8/11 (72.7)	0.17	> 0.9
MRI evidence of acute stroke, no. (%)	45/55 (81.8)	15/21 (71.4)	9/11 (81.8)	5/5 (100)	1/1 (100)	8/8 (100)	0.42	0.05
CT or MRI location of infarction, no. (%)								
Cortical	81/101 (80.2)	29/39 (74.4)	26/28 (92.9)	11/12 (91.7)	0/2 (0)	10/10 (100)	< 0.01	0.09
Subcortical supratentorial	54/101 (53.5)	18/39 (46.2)	15/28 (53.6)	5/12 (41.7)	2/2 (0)	7/10 (70.0)	0.43	0.38
Infratentorial	12/101 (11.9)	7/39 (18.0)	0/28 (0)	1/12 (8.3)	0/2 (0)	1/10 (10.0)	0.12	0.11
**Laboratory testing**								
D-dimer (mcg/mL), median (IQR)	0.78 (0.13–4.9) (*n* = 87)	1.05 (0.21–9.15) (*n* = 36)	0.14 (0.05–0.21) (*n* = 18)	0.61 (0.14–10.08) (*n* = 8)	0.07, 0.49 (*n* = 2)	0.26 (0.13–1.3) (*n* = 6)	0.04	0.02
Admission platelet count (cells/μL), median (IQR)	223 (183–313) (*n* = 143)	242 (201–321) (*n* = 53)	221 (183–289) (*n* = 34)	259 (188–353) (*n* = 15)	198 (106–326) (*n* = 4)	212 (150–281) (*n* = 9)	0.34	0.25
C-reactive protein peak (mg/dL), median (IQR)	11 (3–25) (*n* = 96)	15.8 (6.2–52.7) (*n* = 38)	4.2 (2.4–12.7) (*n* = 23)	12.9 (0.8–74.9) (*n* = 11)	6.2 (0.7–49.9) (*n* = 3)	11.6 (9.5–58.9) (*n* = 4)	0.14	0.05
Admission WBC (cells/μL), median (IQR)	8.4 (6.5–11.9) (*n* = 139)	10.7 (6.8–14.4) (*n* = 53)	8.0 (6.5–10.8) (*n* = 35)	6.8 (5.8–9.5) (*n* = 15)	7.7 (5.7–9.3) (*n* = 4)	6.4 (5.5–6.7) (*n* = 9)	0.05	0.01
Lymphocyte count (cells/μL), median (IQR)	1.2 (0.7–1.7) (*n* = 132)	1.3 (0.7–1.8) (*n* = 47)	1.4 (1.0–1.9) (*n* = 32)	0.7 (0.7–1.1) (*n* = 15)	1.7 (1.2–1.8) (*n* = 4)	1.4 (0.7–1.5) (*n* = 9)	0.05	0.60
**Treatment**								
Intravenous thrombolysis, no. (%)	17/145 (11.7)	4/54 (7.4)	7/35 (20.0)	3/15 (20.0)	0/4 (0)	2/11 (18.2)	0.31	0.12
Endovascular treatment, no. (%)	33/114 (29.0)	8/42 (19.1)	14/26 (53.9)	4/10 (40.0)	0/3 (0)	2/7 (28.9)	0.03	0.03
Endovascular treatment ^d^, no. (%)	33/53 (62.3)	8/17 (47.1)	14/17 (82.4)	4/6 (66.7)	n/a	2/3 (66.7)	0.18	0.11
Acute antithrombotic treatment ^e^, no. (%)							0.01	0.17
Single or dual antiplatelet therapy	66/108 (61.1)	31/43 (72.1)	13/30 (43.3)	12/12 (100)	2/3 (66.7)	6/10 (60.0)		
Therapeutic anticoagulation ^f^	22/108 (20.4)	5/43 (11.6)	11/30 (36.7)	0/12 (0)	1/3 (33.3)	1/10 (10.0)		
None	20/108 (18.5)	7/43 (16.3)	6/30 (20.0)	0/12 (0)	0/3 (0)	3/10 (30.0)		

^a^ Acute ischemic stroke includes suspected or radiographically-confirmed cases, and in patients with multiple possible stroke mechanisms (*n* = 9). Patients with multiple stroke mechanisms were not included in the subsequent columns in order to minimize heterogeneity

^b^ *P*-values indicate categorical comparisons between all stroke subtypes

^c^ *P*-values indicate comparisons between cryptogenic and non-cryptogenic stroke subtypes (including strokes due to multiple possible etiologies)

^d^ Analysis limited to patients with any intracranial occlusion on vascular imaging

^e^ Acute antithrombotic treatment was defined as use of an antithrombotic within 24 h of ischemic stroke diagnosis, or between 24 and 48 h of ischemic stroke diagnosis in patients who received intravenous thrombolysis

^f^ One patient with a stroke due to multiple etiologies was treated with combination antiplatelet (single or dual) with therapeutic anticoagulation

*NIHSS* Denotes National Institutes of Health Stroke Scale, *IQR* Interquartile range, *CT* Computed tomography, *MRI* Magnetic resonance imaging, *HI-1* Denotes hemorrhagic infarction grade 1 (petechial hemorrhage), *HI-2* Hemorrhagic infarction grade 1 (confluent petechiae), *PH-1* Parenchymal hematoma grade 1 (confluent hemorrhage within < 30% of infarct bed), *PH-2* Parenchymal hematoma grade 2 (confluent hemorrhage within > 30% of infarct bed and having mass effect), ICU intensive care unit and mRS modified Rankin Scale

Compared to non-cryptogenic stroke patients, those with cryptogenic strokes had no difference in baseline NIHSS (*p* = 0.53). Patients with cardioembolic stroke had more frequently identified intracranial arterial occlusions and cortical involvement compared to other stroke mechanisms (*p* < 0.05 for both findings), followed by cryptogenic stroke. Admission white blood cell count, C-reactive protein and D-dimer levels were significantly greater for patients with cryptogenic stroke when compared to all other stroke groups, and after multivariable regression with adjustment for all candidate variables significant to *p* < 0.2 (Hispanic ethnicity, congestive heart failure, cortical infarction, white blood cell count and D-dimer), only congestive heart failure was statistically significantly and inversely associated with cryptogenic stroke mechanism (*p* < 0.01; Table 3).

**Table 3:** Multivariable logistic regression model evaluating predictors for cryptogenic stroke in Covid-19 patients

Variable	Univariate analysis		Multivariable analysis	
	OR (95% CI)	*p*-value	OR (95% CI)	*p*-value
**Age, per decade**	0.98 (0.75–1.27)	0.86		
**Male sex**	1.32 (0.64–2.73)	0.45		
**White race**	1.06 (0.46–2.42)	> 0.9		
**Hispanic ethnicity**	1.44 (0.99–2.10)	0.05	*Dropped from model due to non-significance*	
**Medical history**				
Hypertension	0.94 (0.43–2.06)	0.87		
Diabetes mellitus	0.94 (0.46–1.93)	0.87		
Dyslipidemia	0.70 (0.33–1.45)	0.34		
Congestive heart failure	0.35 (0.12–1.02)	0.05	0.15 (0.05–0.44)	< 0.01
Active tobacco use	0.76 (0.21–2.75)	0.68		
Prior stroke	1.86 (0.53–6.47)	0.33		
Chronic renal insufficiency (stage III/IV or dialysis-dependent)	0.61 (0.15–2.50)	0.50		
Chronic obstructive pulmonary disease and/or asthma	0.39 (0.08–1.97)	0.26		
Cancer	2.08 (0.44–9.74)	0.35		
**Baseline NIHSS** ^a^	0.003 (−0.006–0.013)	0.49		
**Imaging**				
Cortical infarction	0.40 (0.14–1.16)	0.09	*Dropped from model due to non-significance*	
Large vessel occlusion	0.95 (0.37–1.95)	0.70		
**Laboratory data** ^a^				
Admission WBC (× 1000/mL)	0.02 (0.002–0.04)	0.03	*Dropped from model due to non-significance*	
Admission lymphocyte count (× 1000/mL)	0.0001 (−0.12–0.12)	> 0.9		
Admission platelet count (× 1000/mL)	0.0004 (−0.0003–0.001)	0.22		
D-dimer (per mcg/mL)	0.009 (0.002–0.017)	0.02	1.73 (0.98–3.04)	0.06
C-reactive protein (per mg/dL)	0.001 (−0.0008–0.003)	0.23		

Variables were entered into the multivariable model if they were significant to *p* < 0.2 in univariate regression (Hispanic ethnicity, congestive heart failure, cortical infarction, elevated d-dimer, elevated admission white blood cell count). Variables were retained if they remained significant to *p* < 0.2. Laboratory values indicate serologic studies collected closest to the time of stroke onset, unless otherwise noted

^a^ Variables in which *β* with 95% confidence interval were used to estimate the effect on the outcome of cryptogenic stroke in univariate analysis. If included in the multivariable model, this effect is displayed as an odds ratio with 95% confidence interval. Multivariable regression model was clustered by site

**Fig. 1:**
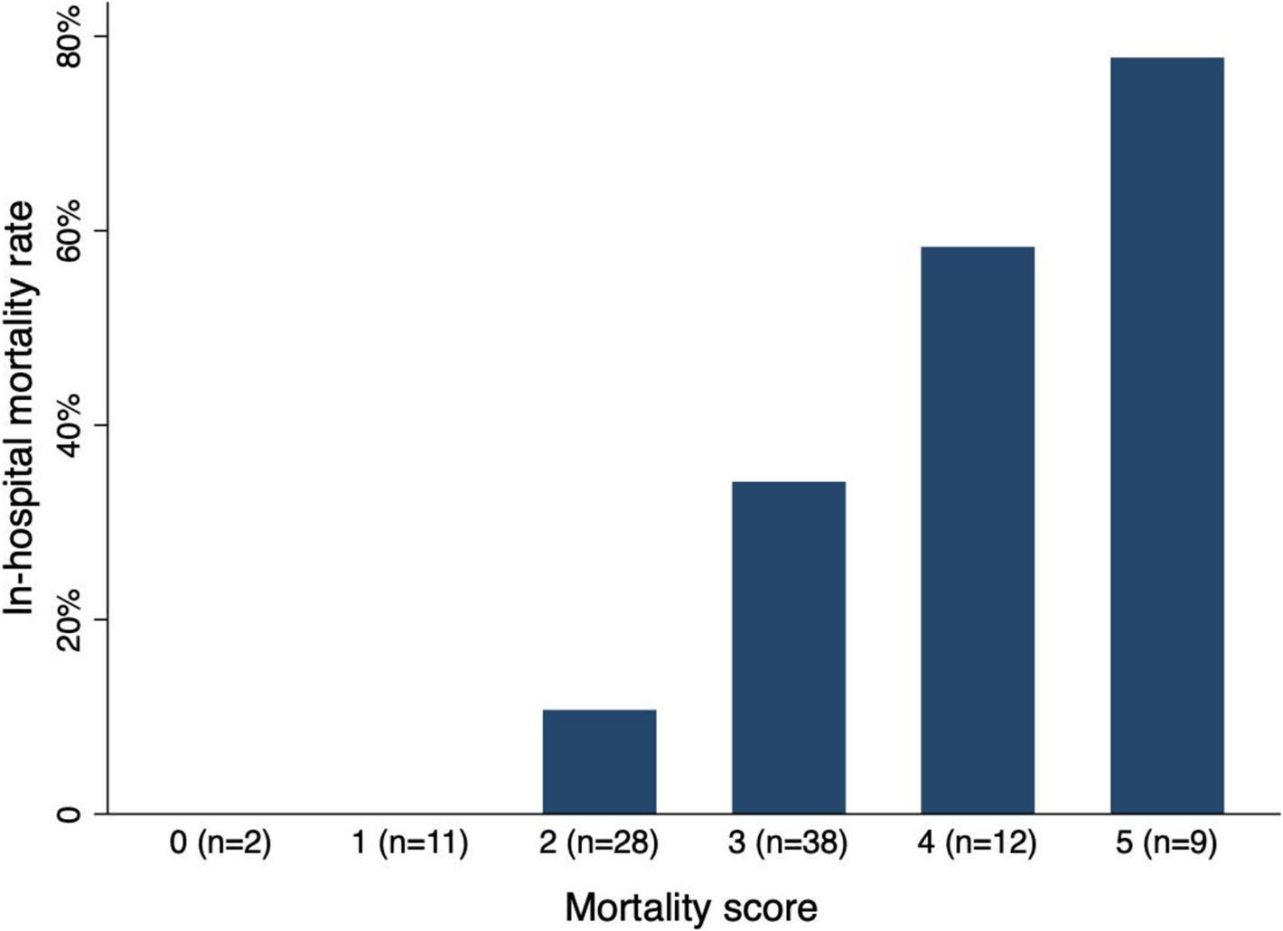
Mortality rate among patients with COVID-19 and stroke according to mortality score. One point was assigned for each clinical factor up to a total of 5 possible points: age 60 or more, male sex, NIHSS 10 or more, history of diabetes, and cryptogenic stroke

Patients with strokes due to cardioembolism and a cryptogenic mechanism had a significantly higher risk of any hemorrhagic transformation when compared to strokes of all other classifications, although event rates for strokes due to ‘other’ causes and small vessel disease were low (*p* = 0.05; Table 4). Patients with cryptogenic stroke were non-significantly more likely to be discharged with greater disability than strokes of other mechanisms (*p* = 0.07), despite having a similar pre-morbid functional status. Furthermore, cryptogenic stroke patients had a significantly higher risk of in-hospital mortality when compared to strokes of all other mechanisms (OR 2.27, 95%CI 1.01–5.08, *p* = 0.05). In the backward stepwise regression model for in-hospital mortality, cryptogenic stroke remained independently associated with in-hospital mortality (aOR 5.16, 95%CI 1.41–18.87, *p* = 0.01). In that model, older age (aOR 2.05 per decade, 95%CI 1.35–3.11, *p* < 0.01), higher baseline NIHSS (aOR 1.12, 95%CI 1.02–1.21, *p* = 0.01), and history of diabetes (aOR 6.89, 95%CI 1.02–46.76, *p* = 0.05) remained independently associated with mortality, whereas male sex was slightly but non-significantly associated with mortality (aOR 2.39, 95%CI 0.69–8.28, *p* = 0.17; supplementary Table 1). Neither intravenous thrombolysis (OR 0.82, 95%CI 0.27–2.52, *p* = 0.73) nor thrombectomy (OR 0.97, 95%CI 0.40–2.34, *p* = 0.95) were associated with mortality in univariate modeling. A simple risk score was derived from the five variables from the backward stepwise regression model by assigning 1-point each in order to estimate the risk of in-hospital mortality. Additional points showed an incrementally greater probability of mortality (Fig. 1), with the total score having a strong discriminatory power to predict in-hospital mortality (area under the curve 0.79, 95%CI 0.70–0.88). Three or more points indicated an 88.9% sensitivity and 53.0% specificity for the outcome of in-hospital mortality.

**Table 4:** Outcome measures

	All patients acute ischemic stroke^a^ (*n* = 156)	Cryptogenic stroke (*n* = 55)	Cardioembolic stroke (*n* = 35)	Large vessel stroke (*n* = 15)	Small vessel stroke (*n* = 4)	Other (*n* = 11)	*p*-value^b^	*p*-value^c^
Hemorrhagic transformation of infarction, no. (%)	17/98 (17.4)	6/42 (14.3)	3/26 (11.5)	0/8 (0)	0/4 (0)	4/7 (57.1)	0.05	0.55
Grade of hemorrhage							> 0.9	0.82
HI-1	7/17 (41.2)	3/6 (50.0)	1/3 (33.3)	n/a	n/a	2/4 (50.0)		
HI-2	2/17 (11.8)	1/6 (16.7)	0/3 (0)	n/a	n/a	1/4 (25.0)		
PH-1	4/17 (23.5)	1/6 (16.7)	1/3 (33.3)	n/a	n/a	1/4 (25.0)		
PH-2	4/17 (23.5)	1/6 (16.7)	1/3 (33.3)	n/a	n/a	0/4 (0)		
Symptomatic ICH^d^, no. (%)	1/14 (7.1)	1/4 (25.0)	0/3 (0)	n/a	n/a	0/4 (0)	> 0.9	> 0.9
Transfer to ICU, no. (%)	69/145 (47.6)	26/52 (50.0)	11/33 (33.3)	10/15 (66.7)	0/4 (0)	10/11 (90.9)	< 0.01	0.36
Intubation, no. (%)	51/139 (36.7)	17/47 (36.2)	7/32 (21.9)	4/15 (26.7)	0/4 (0)	7/11 (63.6)	0.08	0.56
Discharge disposition, no. (%)							0.57	0.55
Home	34/136 (25.0)	11/50 (22.0)	9/29 (31.0)	4/14 (28.6)	2/4 (50.0)	5/11 (45.5)		
Acute inpatient rehabilitation	18/136 (13.2)	8/50 (16.0)	3/29 (10.3)	1/14 (7.1)	1/4 (25.0)	2/11 (18.2)		
Skilled nursing facility or subacute rehabilitation	20/136 (14.7)	5/50 (10.0)	6/29 (20.7)	4/14 (28.6)	0/4 (0)	2/11 (18.2)		
Long-term acute care	5/136 (3.7)	2/50 (4.0)	1/29 (3.5)	2/14 (14.3)	0/4 (0)	0/11 (0)		
Other acute care facility	7/136 (5.2)	3/50 (6.0)	3/29 (10.3)	0/14 (0)	0/4 (0)	0/11 (0)		
Hospice	2/136 (1.5)	1/50 (2.0)	0/29 (0)	0/14 (0)	0/4 (0)	1/11 (9.1)		
Expired during hospitalization	53/139 (38.1)	20/50 (40.0)	7/29 (24.1)	3/14 (21.4)	1/4 (25.0)	1/11 (9.1)		
Modified Rankin Scale at discharge, median (IQR)	4 (2–6) (*n* = 139)	5 (3–6) (*n* = 51)	4 (2–5) (*n* = 32)	4 (2–5) (*n* = 14)	1 (0–4) (*n* = 4)	3 (2–4) (*n* = 10)	0.20	0.07
Discharge mRS 0–2, no. (%)	35/139 (25.2)	11/51 (21.6)	11/32 (34.4)	4/14 (28.6)	3/4 (75.0)	3/10 (30.0)	0.21	0.14
In-hospital mortality, no. (%)	53/139 (38.1)	20/50 (40.0)	7/29 (24.1)	3/14 (21.4)	1/4 (25.0)	1/11 (9.1)	0.18	0.05

^a^ Acute ischemic stroke includes suspected or radiographically-confirmed cases, and in patients with multiple possible stroke mechanisms (*n* = 9). Patients with multiple stroke mechanisms were not included in the subsequent columns in order to minimize heterogeneity

^b^ *P*-values indicate categorical comparisons between all stroke subtypes

^c^ *P*-values indicate comparisons between cryptogenic and non-cryptogenic stroke subtypes (including strokes due to multiple possible etiologies)

^d^ Symptomatic ICH defined as a PH-2 grade hematoma with worsening of the NIHSS by 4 or more points

## Discussion

In this observational cohort of more than 14,000 COVID-19 patients treated at 31 hospitals in 4 countries, we found a high proportion of strokes among patients with COVID-19 were attributed to a cryptogenic mechanism (42%). This finding is concordant with the growing literature indicating a high prevalence of cryptogenic stroke in COVID-19, [6, 13, 20, 21] with increasing data suggesting the novel human coronavirus may be a novel stroke mechanism. In this study, a cryptogenic stroke diagnosis was made at the judgment of the treating vascular neurologist and was inclusive of strokes with incomplete workup. Use of more stringent trial criteria for “cryptogenic stroke” (which requires thorough evaluation for cardiac and vascular causes of cerebral infarction) in the present study would certainly strengthen the observed association implicating COVID-19 as a potentially unique stroke mechanism. However, we did not centrally adjudicate vascular, echocardiographic, or parenchymal imaging in order to confirm the diagnosis of cryptogenic infarction. Our determination of cryptogenic stroke is congruent with many published reports which did not report a detailed methodology for determination of cryptogenic mechanism, [22] did not require intracranial vascular imaging, [23] or included patients for whom workup could not be completed [20]. Therefore, we do not believe the clinical determination of cryptogenic stroke to be a significant limitation of this analysis.

Perhaps the most important observation in this cohort is the relationship between stroke mechanism and in-hospital mortality. When compared to other discharge dispositions, death was the most common singular discharge disposition in this cohort of stroke patients. When death was evaluated as a bivariate outcome, having a cryptogenic stroke mechanism was significantly and independently predictive of in-hospital mortality. The high mortality rate is consistent with other cohorts [6, 13, 20] and adds to the results of one recent systematic review and meta-analysis demonstrating a 5-fold increase in in-hospital mortality among COVID-19 stroke patients when compared to their contemporary noninfected or historical controls [21]. It should be noted that patients in the present cohort contributed to this meta-analysis by Katsanos and colleagues; however the patients in the present study did not contribute to the comparison of in-hospital mortality rates among COVID-19 stroke patients and COVID-19-negative controls.

In our study, admission white blood cell count, c-reactive protein, and D-dimer levels were significantly higher for patients later diagnosed with cryptogenic stroke. It has been described that SARS-CoV-2 infection is linked to a prothrombotic state with elevated D-dimer levels [24]. This finding also suggests that hypercoagulability could be one of the causes underlying this high proportion of cryptogenic strokes [25]. The high incidence of intracranial occlusion (46%) and cortical (74.4%) strokes also suggest a disproportionate number of patients with embolic strokes who suffer from COVID-19. These findings are in keeping with recently published observations from a New York hospital system [20]. Together, these observations validate the relationship between a significant inflammatory and/or prothrombotic state and clinically significant stroke that is unrelated to other traditional stroke risk factors (e.g., cervical artery atherosclerosis or atrial fibrillation). Furthermore, the higher mortality rate observed in patients with cryptogenic stroke speaks to the severity of COVID-19 experienced by these patients. On the other hand, diagnostic workup for patients in this study (and in other studies) [20, 22, 23] may not have been completed for some patients with COVID-19 in whom care was being withdrawn or who expired soon before workup could have been completed. This might have confounded our observation of a high rate of cryptogenic stroke and associated mortality, as reported in one prior study [11].

Due to the strong relationship between cryptogenic stroke and early mortality, we derived a simple risk score for mortality that could serve as a useful tool in hospitalized stroke patients with COVID-19 when making long-term plans of care. Patients in this cohort who met each of the 5 criteria (age, male sex, diabetes, NIHSS 10+, cryptogenic stroke) were at an 80% chance of in-hospital mortality, whereas those who met at least 3 criteria were still more likely to die than to survive hospitalization. However, this score is not without limitations, and it certainly warrants external validation. First, the NIHSS is not a specific indicator of stroke severity in critically ill patients with COVID-19, as it can be confounded by sedation. Second, a patient’s history of diabetes does not reflect how well or poorly controlled the medical condition is, and we did not evaluate the risk of mortality based on markers of diabetic control (e.g., hemoglobin A1c) or duration of disease. Further, the diagnosis of a stroke as ‘cryptogenic’ may be contingent upon which testing was or was not pursued to evaluate the proximate cause of stroke in a critically ill patient. As in previously published reports of cryptogenic stroke in COVID-19 patients [20], patients with a high probability of dying after a severe stroke may not have undergone a more comprehensive workup, and therefore could have been prematurely classified as ‘cryptogenic’. Determination of a stroke being cryptogenic on the basis of incomplete workup due to high probability of imminent death or pursuit of comfort measures may have falsely contributed to its association with in-hospital mortality. That said, as we have previously reported [7], the median delay from stroke onset to death was 4 days in this cohort, (IQR 1–10 days) which suggests that echocardiographic and vascular imaging could have been performed in advance of expiration.

While this study is one of the largest observational cohorts of consecutive COVID-19 patients with cerebrovascular complications, it is limited by its retrospective nature and the completeness of data elements that were abstracted from the medical record. The imaging findings were not centrally adjudicated. Selection and interpretation of diagnostic tests were made at the discretion of the treating physician, and the derivation of a simplified mortality score warrants external validation. That said, our data reflect a large cohort of consecutive patients evaluated at many sites and reflect the diverse experiences in care of patients with stroke and COVID-19.

## Conclusions

Although the overall incidence rate of acute ischemic stroke in hospitalized patients with COVID-19 is small, there is a higher than expected proportion of patients with cryptogenic stroke. Furthermore, patients with cryptogenic stroke and COVID-19 are at a significantly greater risk of early mortality when compared to patients with COVID-19 and known, traditional stroke mechanisms. The relationship between cryptogenic stroke in COVID-19 and mortality may be driven by more severe inflammatory or prothrombotic disease in COVID-19. A simple 5-point score may be useful in clinical decision making for patients at a low risk of short-term survival, however this score requires external validation.

## Supplementary Information

**Additional file 1 Supplementary Table 1**. **Supplementary Figs. 1 and 2**

## Data Availability

The datasets used and/or analyzed during the current study are available from the corresponding author on reasonable request.
