# Supplementary material for: Stroke etiologies in patients with COVID-19: the SVIN COVID-19 multinational registry
Source: BMC Neurol. 2021 Jan 30;21:43. doi: 10.1186/s12883-021-02075-1 (PMC7846488; doi:10.1186/s12883-021-02075-1)
Supplement: Supplementary file 1 — Additional file 1 Supplementary Table 1. Supplementary Figs. 1 and 2 [file 12883_2021_2075_MOESM1_ESM.docx]

**Supplementary Table 1. Univariate predictors of in-hospital mortality among stroke patients with COVID-19.**

| Variable | Univariate analysis | | Multivariable analysis | |
| --- | --- | --- | --- | --- |
|  | OR (95% CI) | p-value | OR (95% CI) | p-value |
| **Age, per decade** | 1.38 (1.03-1.85) | 0.03 | 2.05 (1.35-3.11) | <0.01 |
| **Male sex** | 2.37 (1.13-4.98) | 0.02 | 2.39 (0.69-8.28) | 0.17 |
| **White race** | 0.67 (0.29-1.50) | 0.33 |  |  |
| **Hispanic ethnicity** | 1.18 (0.81-1.70) | 0.39 |  |  |
| **Medical history** |  |  |  |  |
| Hypertension | 1.63 (0.71-3.74) | 0.25 |  |  |
| Diabetes mellitus | 1.64 (0.82-3.30) | 0.17 | 6.89 (1.02-46.76) | 0.05 |
| Dyslipidemia | 1.00 (0.47-2.11) | >0.9 |  |  |
| Congestive heart failure | 1.13 (0.45-2.84) | 0.79 |  |  |
| Active tobacco use | 2.08 (0.68-6.33) | 0.20 | *Dropped from model due to non-significance* |  |
| Prior stroke | 1.13 (0.35-3.67) | 0.84 |  |  |
| Chronic renal insufficiency (stage III/IV or dialysis-dependent) | 0.86 (0.27-2.68) | 0.79 |  |  |
| Chronic obstructive pulmonary disease and/or asthma | 1.67 (0.48-5.82) | 0.42 |  |  |
| Cancer | 2.70 (0.58-12.69) | 0.21 |  |  |
| **Baseline NIHSS** ^a^ | 0.02 (0.008-0.03) | <0.01 | 1.12 (1.02-1.21) | 0.01 |
| **Imaging** |  |  |  |  |
| Cortical infarction ^b^ | 0.29 (0.10-0.86) | 0.03 |  |  |
| Large vessel occlusion ^b^ | 2.01 (0.87-4.67) | 0.10 | *Dropped from model due to non-significance* |  |
| **Laboratory data** ^a^ |  |  |  |  |
| Admission WBC (x 1,000/mL) | 0.008 (-0.003-0.02) | 0.14 | *Dropped from model due to non-significance* |  |
| Admission lymphocyte count (x 1,000/mL) | -0.16 (-0.28- -0.03) | 0.02 |  |  |
| Admission platelet count (x 1,000/mL) | -0.001 (-0.001- -0.0001) | 0.02 | *Dropped from model due to non-significance* |  |
| D-dimer (per mcg/mL) | 0.003 (-0.004-0.010) | 0.45 |  |  |
| C-reactive protein (per mg/dL) | 0.00005 (-0.002-0.002) | 0.96 |  |  |
| **Cryptogenic stroke diagnosis** | 2.27 (1.01-5.08) | 0.05 | 5.16 (1.41-18.87) | 0.01 |
| Intravenous Thrombolysis | 0.82 (0.27-2.52) | 0.73 | *Dropped from model due to non-significance* |  |
| Mechanical Thrombectomy | 0.97 (0.40-2.34) | 0.95 | *Dropped from model due to non-significance* |  |

Variables were entered into the multivariable model if they were significant to p<0.2 in univariate regression (Hispanic ethnicity, congestive heart failure, elevated d-dimer, elevated admission white blood cell count). Variables were retained if they remained significant to p<0.2. Laboratory values indicate serologic studies collected closest to the time of stroke onset, unless otherwise noted.

^a^ Variables in which *β* with 95% confidence interval were used to estimate the effect on the outcome of cryptogenic stroke in univariate analysis. If included in the multivariable model, this effect is displayed as an odds ratio with 95% confidence interval. Multivariable regression model was clustered by site.

^a^ Variables “cortical infarction” and “large vessel occlusion” were collinear. Therefore, only the latter variable was included in the multivariable model.
